# Prognosis and Biological Behavior of Gastric Signet-Ring Cell Carcinoma Better or Worse: A Meta-Analysis

**DOI:** 10.3389/fonc.2021.603070

**Published:** 2021-06-30

**Authors:** Shuai Zhao, Ling Lv, Kai Zheng, Yu Tian, Jian-Chun Zheng, Cheng-Gang Jiang

**Affiliations:** ^1^ Department of Surgical Oncology and General Surgery, Key Laboratory of Precision Diagnosis and Treatment of Gastrointestinal Tumors, Ministry of Education, The First Affiliated Hospital of China Medical University, Shenyang, China; ^2^ Department of Thoracic Surgery, The First Affiliated Hospital of China Medical University, Shenyang, China

**Keywords:** gastric neoplasm, signet-ring cell carcinoma, prognosis, meta-analysis, biological behavior

## Abstract

**Background:**

The clinical pathology of gastric signet-ring cell carcinoma (SRC) is still unclear. This meta-analysis was performed to evaluate the difference in biological behavior and prognosis between SRC and non-signet ring cell carcinoma (NSRC).

**Methods:**

A total of 58 eligible studies were analyzed using RevMan and other auxiliary software. Biological behaviors were compared based on odds ratio (OR) and mean difference (MD). Hazards ratio (HR) was calculated for prognosis based on Kaplan–Meier curves.

**Results:**

Totally, 28,946 SRC patients were compared with 81,917 NSRC patients. Compared with NSRC patients, lower male: female ratio (OR = 0.53, *P* < 0.01), younger age (MD = −4.89, *P* < 0.01), more middle location (OR = 1.64, *P* < 0.01), more depressed type at early stage (OR = 1.31, *P* < 0.05), higher incidence of Borrmann type IV (OR = 1.96, *P* < 0.01), less lymph node metastasis at early stage (OR = 0.78, *P* < 0.05), better prognosis at early stage (HR = 0.59, *P* < 0.01), and worse prognosis at advanced stage (HR = 1.19, *P* < 0.01) were associated with SRC patients.

**Conclusion:**

The prognosis of SRC at early stage is better than other types of gastric cancer, while that of SRC at advanced stage is relatively poorer.

## Introduction

Signet-ring cell carcinoma (SRC) is associated with unique histological features based on microscopic observation of the tumor cells rather than on biological behavior. Gastric SRC has been categorized as the “undifferentiated type” by Sugano et al. (1), the “diffused type” by Lauren et al. (2), the “infiltrative type” by Ming et al. (3), and “high grade type” by UICC. Several studies have shown that SRC is associated with a high rate of peripheral metastasis and poor prognosis (4–9); however, a few studies have indicated that SRC has a better outcome than other types of gastric cancer (GC) (10, 11). Meanwhile, several studies have demonstrated that the difference in survival rates between SRC and non-signet ring cell carcinoma (NSRC) is statistically insignificant (12, 13). Additionally, multiple studies have also indicated that early stage gastric SRC has a higher five-year survival rate than NSRC (14, 15). Here, we aimed to elucidate the difference in biological behavior between SRC and NSRC.

This meta-analysis compared the biological behavior and prognosis between SRC and NSRC patients, including gender, tumor location, lymph node metastasis (LNM), age, chemotherapy, tumor size, macroscopic type, and overall survival.

## Methods

### Population

All patients were diagnosed with GC.

### Intervention and Comparator

#### Exposure Group

Patients who were diagnosed with SRC based on pathological analyses.

#### Control Group

Patients who were diagnosed with NSRC based on pathological analyses.

### Outcomes

Biological behavior and prognosis.

### Study Design

This meta-analysis complied with the PRISMA statement. All the included studies were primary research studies. There were no language restrictions.

### Search Strategy

The Web of Science, PubMed, and Embase databases were searched from initiation until November 2020 as follows: “((“gastric” [Title/Abstract] OR “stomach” [Title/Abstract]) AND (((“cancer” [Title/Abstract] OR “tumor” [Title/Abstract]) OR “carcinoma” [Title/Abstract]) OR “neoplasm” [Title/Abstract])) AND ((“signet ring cell” [Title/Abstract] OR “signet-ring cell” [Title/Abstract]) OR “signet cell” [Title/Abstract])”, including both published and unpublished articles. There were no language restrictions. The articles were retrieved by more than three independent investigators and compiled.

### Inclusion and Exclusion Criteria

The criteria for study enrollment were as follows: [1] Based on the WHO classification, SRC was classified when more than 50% cancer cells were predominantly SRC. [2] All studies related to the prognosis and biological behavior of gastric SRC were included. [3] All studies that showed differences in the biological behavior and prognosis between SRC and NSRC were included. [4] All the included studies were primary research articles. [5] If the same research team reported multiple studies during the same time period, only the latest article or that with complete data was included.

Studies without full text or efficacious data were excluded. Additionally, case reports and editorials were not included.

### Data Extraction and Quality Assessment

The following data were extracted from the included studies: publication year, name of the first author, country of author, sample size, and clinicopathological features (*e.g.*, sex ratio, mean age, tumor location, tumor size, chemotherapy, macroscopic type, LNM, and overall survival). However, owing to insufficient data, other variables of clinicopathological features (*e.g.*, venous invasion, peritoneal dissemination, and ulceration) were not extracted or analyzed. Engauge Digitizer 4.1 was employed to distinguish the survival curve and extract hazard ratio (HR) of overall survival (data not shown).

### Statistical Analysis

All data were analyzed using Review manager 5.3 and Stata 12.0. Heterogeneity was detected by chi-square test. *P*-value >0.10 was considered as homogeneous, otherwise as heterogeneous. Moreover, the I^2^ index was used to assess heterogeneity, and I^2^ >50% was considered as statistically significant. For homogeneous affirmation, the fixed effects model was selected; otherwise, a random effects model was adopted. The odds ratio (OR), mean difference (MD), and hazard ratio (HR) were calculated, and publication bias was assessed by Egger’s test.

## Result

### Search Result

**Figure 1:**
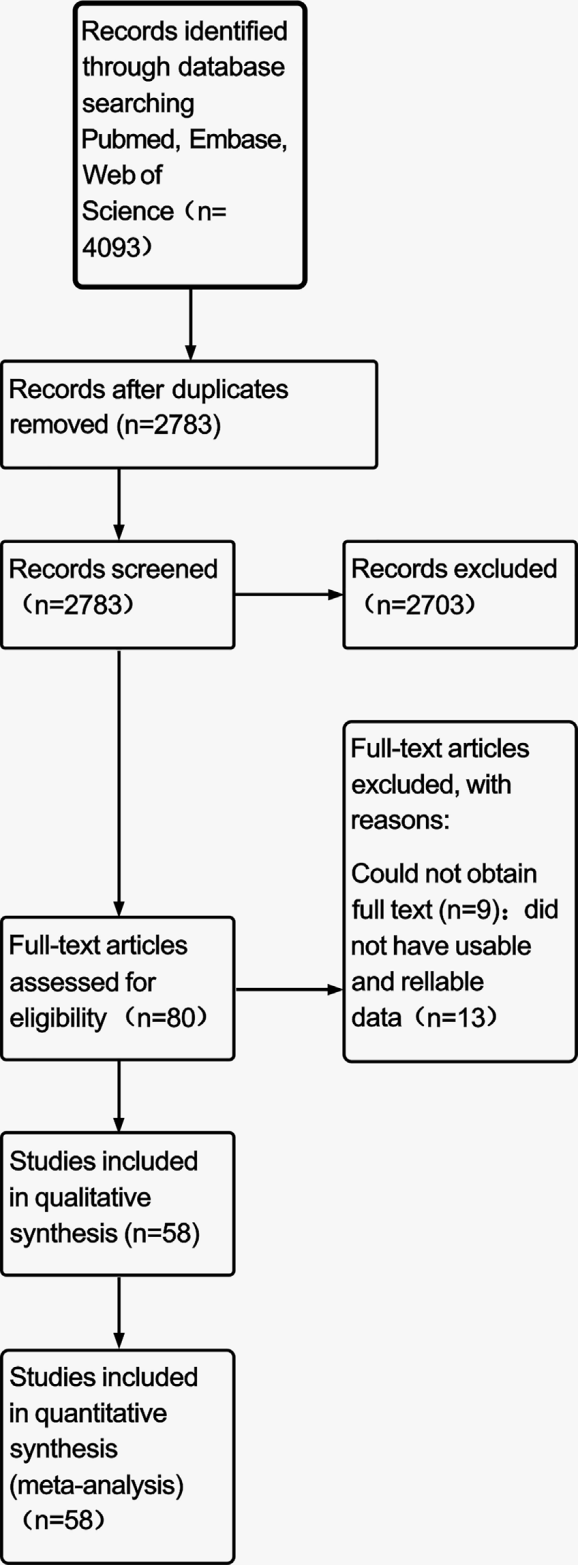
Schematic of the search and selection of the studies.

A total of 4,093 studies were retrieved from PubMed, Web of Science, and Embase. After reading the abstracts, we further assessed the full text of 80 studies; we could not obtain the full text for nine studies; 13 contained no usable and reliable data. Finally, 58 eligible studies (5–62) were included in this meta-analysis (**Figure 1**), among which 31 reported the entire period of patients with SRC or NSRC, 25 focused on early GC, and two reported advanced GC regarding both SRC and NSRC (**Table 1**). The study population of SRC (28,946) was much smaller than that of NSRC (81,917).

**Table 1:** Information of the included studies.

Author	Year	Country	Research stage	Total	SRC	NSRC	NOS
Aihara (16)	2006	Japan	Early stage	150	76	74	6
Anh (17)	2020	Korea	Whole period	460	200	260	6
Bozkaya (18)	2017	Turkey	Whole period	193	142	51	6
Cai (19)	2017	China	Whole period	2,980	133	2,847	6
Chen J (20)	2018	China	Whole period	241	62	179	6
Chen JN (21)	2020	China	Early stage	1,107	203	904	7
Chiu (12)	2011	China	Whole period	2,439	505	1,934	8
Chon (22)	2017	Korea	Whole period	7,667	1,646	6,021	7
Cui (23)	2015	China	Early stage	1,447	288	1,159	7
Gronnier (24)	2013	France	Early stage	421	104	317	7
Guo CG (25)	2015	China	Early stage	720	198	522	6
Guo S (26)	2019	China	Whole period	16,482	3715	12,767	7
Ha (14)	2008	Korea	Early stage	641	388	253	7
Huang (27)	2020	China	Whole period	441	181	260	7
Huh (28)	2013	Korea	Early stage	720	198	522	6
Hyung (29)	2002	Korea	Early stage	933	263	670	7
Imamura (30)	2016	Japan	Early stage	746	190	556	7
Jiang (13)	2011	China	Whole period	2,315	211	2,104	7
Jin (31)	2015	Korea	Early stage	1,105	227	878	7
Kao (32)	2019	China	Whole period	2,152	570	1,582	7
Kim BS (33)	2014	Korea	Early stage	2,050	345	1,705	7
Kim DY (34)	2004	Korea	Whole period	2,358	204	2,154	8
Kim HM (35)	2011	Korea	Early stage	707	419	288	6
Kim JP (36)	1994	Korea	Whole period	3,399	450	2,949	7
Kim YH (37)	2016	Korea	Early stage	1,471	1,046	425	6
Kong (38)	2016	China	Whole period	480	90	390	7
Kunisaki (39)	2004	Japan	Whole period	1,113	174	939	8
Kwon (40)	2014	Korea	Whole period	769	108	661	6
Lai (41)	2016	China	Early stage	2,873	745	2,128	7
Lee HH (10)	2012	Korea	Whole period	1,322	320	1,002	7
Lee IS (42)	2017	Korea	Early stage	1,161	652	509	6
Lee JH (43)	2010	Korea	Whole period	1,362	448	914	7
Lee SH (44)	2015	Korea	Early stage	696	114	582	7
Li C (45)	2007	Korea	Advanced stage	4,759	662	4,097	7
Li H (46)	2016	China	Early stage	81	7	74	6
Liu (5)	2015	China	Whole period	1,464	138	1,326	7
Lu (47)	2016	China	Whole period	2,199	354	1,845	7
Maehara (11)	1992	Japan	Whole period	1,500	51	1,449	8
Nakamura (48)	2019	Japan	Early stage	314	209	105	6
Nam (49)	2010	Korea	Early stage	2,518	720	1,798	7
Otsuji (50)	1998	Japan	Whole period	1,498	154	1,344	7
Park (51)	2008	Korea	Whole period	2,275	251	2,024	7
Piessen (6)	2009	France	Whole period	159	59	100	7
Postlewait (7)	2015	America	Whole period	768	312	456	6
Shim (52)	2014	Korea	Whole period	2,643	377	2,266	7
Taghavi (53)	2012	America	Whole period	10,246	2,666	7,580	8
Tang (54)	2020	China	Whole period	6,017	5,265	752	7
Tong (55)	2011	China	Early stage	422	102	320	7
Voron (8)	2016	France	Whole period	1,799	899	900	7
Wang JM (56)	2010	China	Early stage	103	38	65	7
Wang Z (15)	2015	China	Early stage	334	115	219	7
Yokota (9)	1998	Japan	Whole period	683	93	590	7
Yoon (57)	2016	Korea	Early stage	3,058	930	2,128	7
Zhang (58)	2010	China	Whole period	1,439	218	1,221	8
Zhao (59)	2020	China	Whole period	1,891	235	1,656	8
Zhu (60)	2020	China	Early stage	508	278	230	6
Zou (61)	2020	China	Early stage	323	154	169	6
Zu (62)	2014	China	Advanced stage	741	44	697	6

NOS, Newcastle-Ottawa scale.

Quality assessment was conducted with Newcastle–Ottawa scale based on three indexes (a maximum of nine points): selection, comparability, and exposure. Among the 58 included studies, 17 scored six points, 34 scored seven points, and seven scored eight points. Based on the threshold of six points, all the studies were eligible.

### Clinicopathological Characteristics

**Figure 2:**
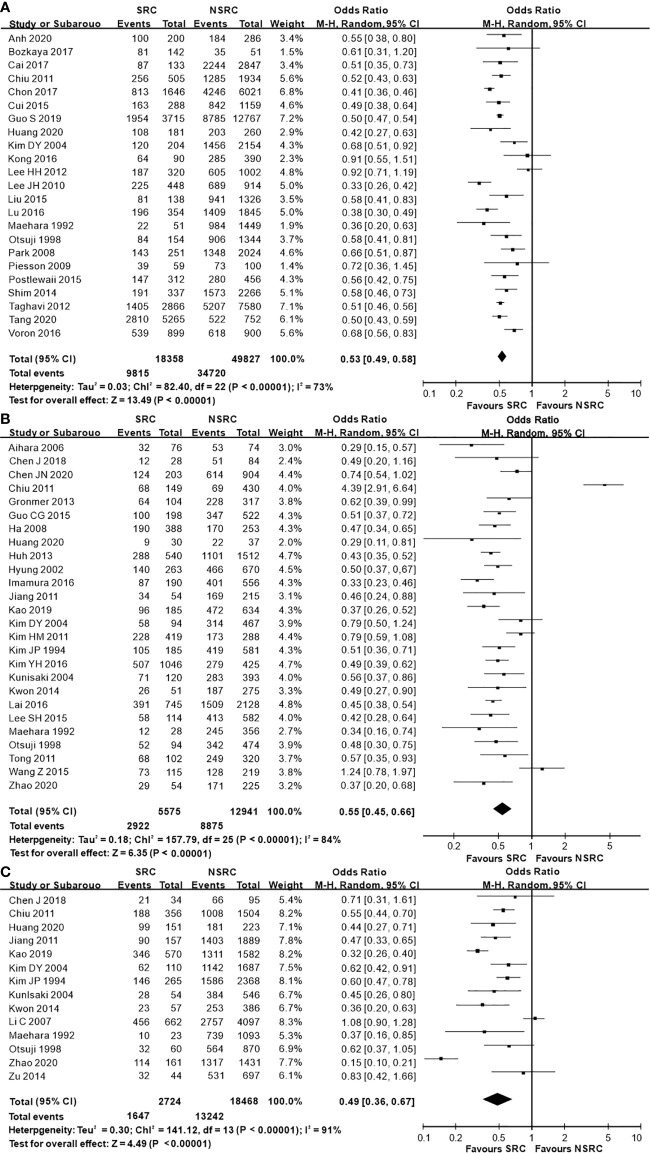
Forest plot displaying the results of the meta-analysis. **(A)** Odds ratio for the male ratio of patients with SRC and NSRC. **(B)** Odds ratio for male ratio at early stage. **(C)** Odds ratio for male ratio at advanced stage.

**Figure 3:**
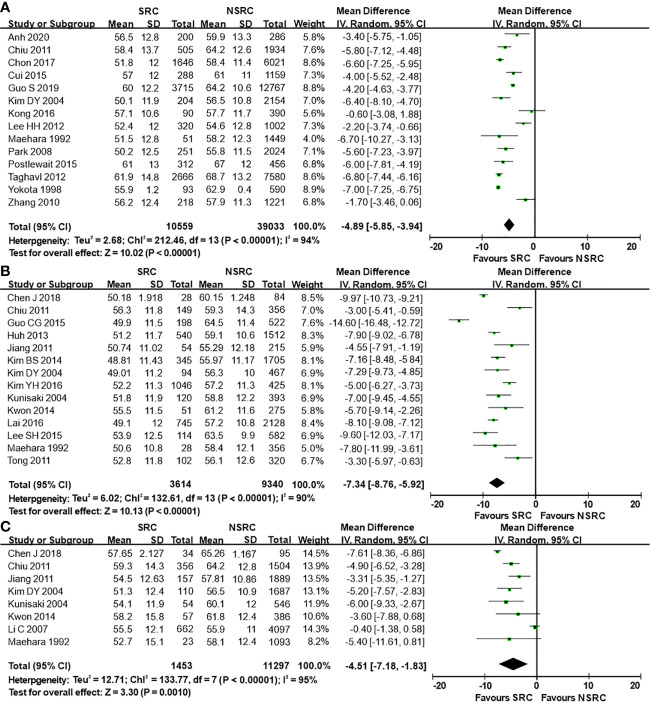
Forest plot displaying the results of meta-analysis. **(A)** Mean difference for mean age of patients with SRC and NSRC. **(B)** Mean difference for mean age at early stage. **(C)** Mean difference for mean age at advanced stage.

**Figure 4:**
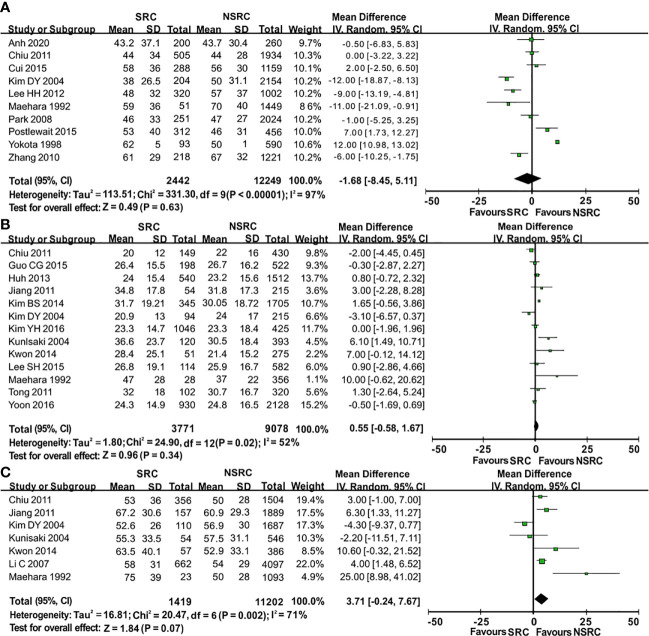
Forest plot displaying the results of meta-analysis. **(A)** Mean difference for tumor size of patients with SRC and NSRC. **(B)** Mean difference for tumor size at early stage. **(C)** Mean difference for tumor size at advanced stage.

**Figure 5:**
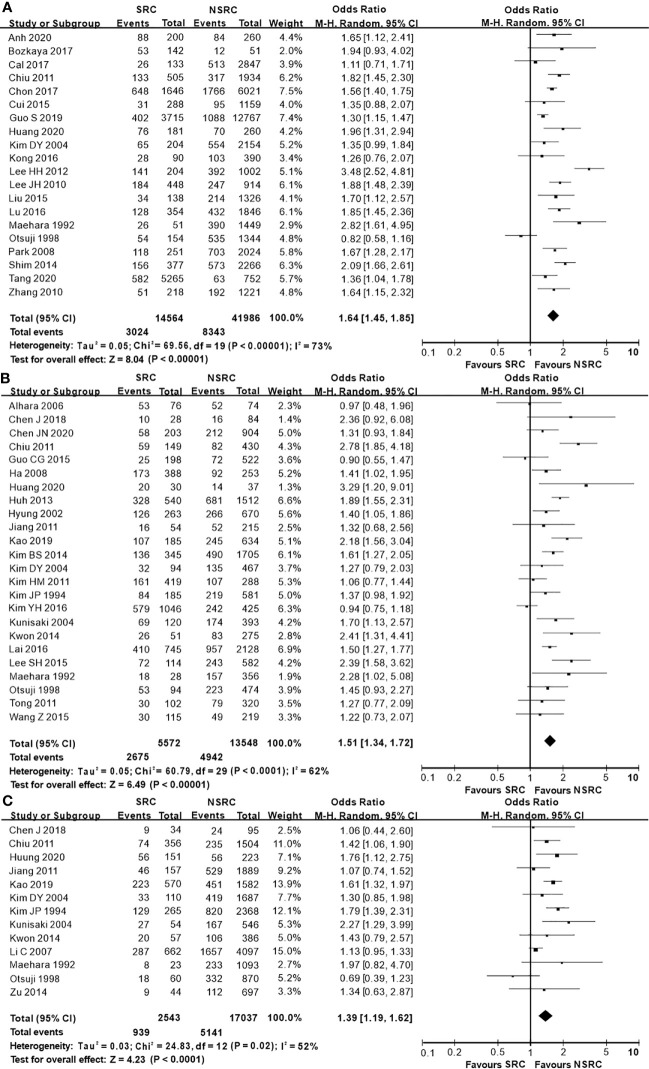
Forest plot displaying the results of meta-analysis. **(A)** Odds ratio for middle location of patients with SRC and NSRC. **(B)** Odds ratio for middle location at early stage. **(C)** Odds ratio for middle location at advanced stage.

**Figure 6:**
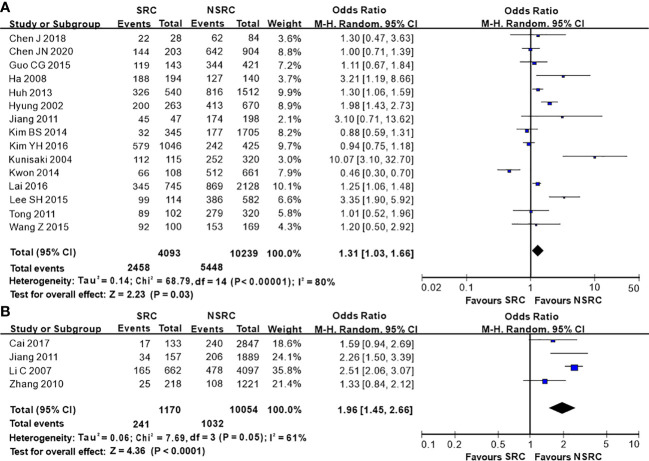
Forest plot displaying the results of meta-analysis. **(A)** Odds ratio for depressed type of patients with SRC and NSRC at early stage. **(B)** Odds ratio for Borrmann IV at advanced stage. (DT, depressed type; B-4, Borrmann IV).

**Figure 7:**
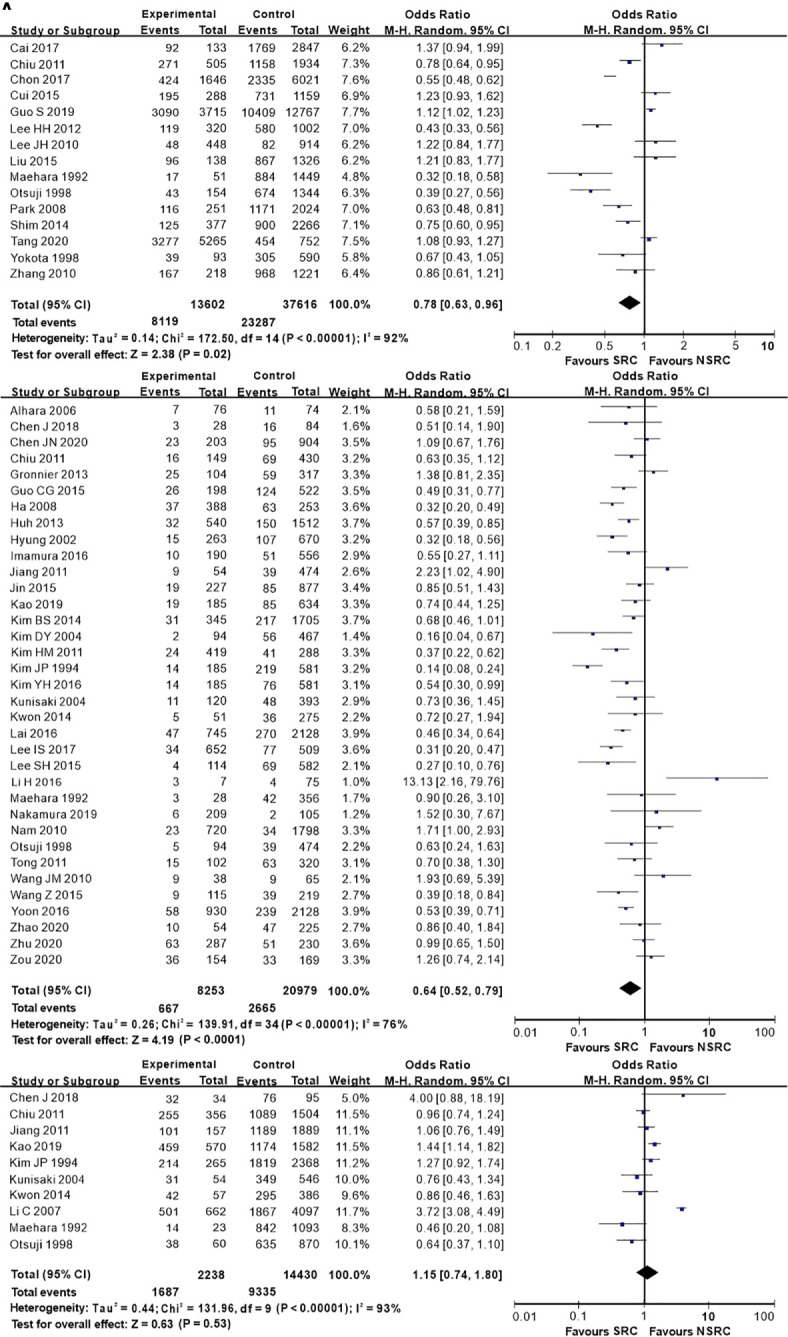
Forest plot displaying the results of meta-analysis. **(A)** Odds ratio for lymph node metastasis of patients. **(B)** Odds ratio for lymph node metastasis at early stage. **(C)** Odds ratio for lymph node metastasis at advanced stage. (LNM, lymph node metastasis).

**Figure 8:**
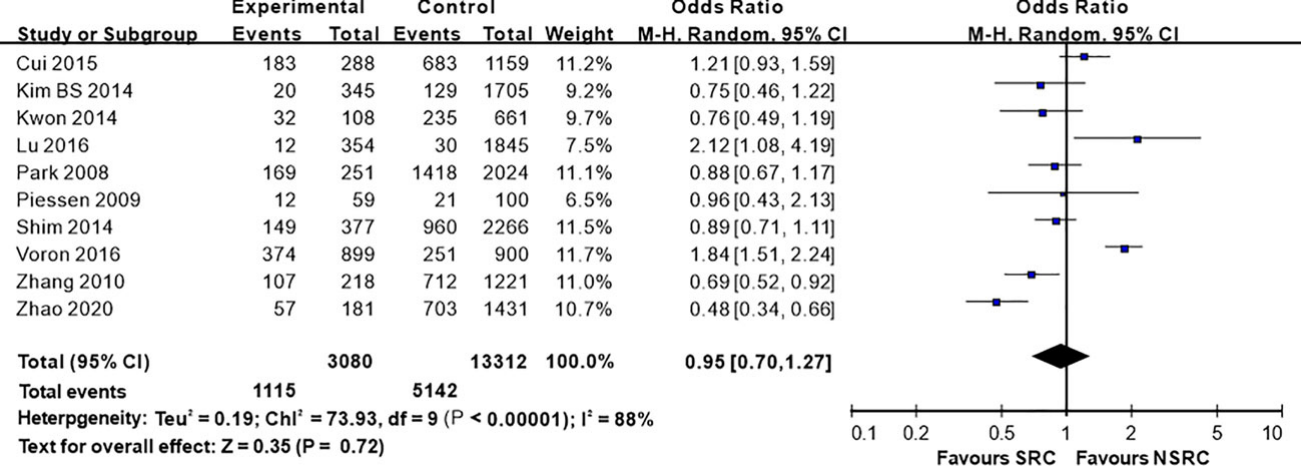
Forest plot displaying the results of meta-analysis. Odds ratio for chemotherapy rate of patients with SRC and NSRC.

The percentage of male patients of SRC was substantially less than that of NSRC (OR = 0.53, 95%CI = 0.49–0.58, *P* < 0.01; **Figure 2**). The mean age of SRC patients was substantially younger than that of NSRC patients, at both early and advanced stages (MD = −4.89, 95%CI = −5.85–3.94, *P* < 0.01; **Figure 3**). No statistical difference in tumor size of SRC between SRC and NSRC was observed, irrespective of early GC (EGC) or advanced GC (AGC) (total: MD = −1.68, 95%CI = −8.48–5.11, *P* = 0.63; EGC: MD = 0.55, 95%CI = −0.58–1.67, *P* = 0.34; AGC: MD = 3.71, 95%CI = −0.24–7.67, *P* = 0.07; **Figure 4**). SRC was found to potentially occur at the middle location of the stomach, irrespective of EGC or AGC (OR = 1.64, 95%CI = 1.45–1.85, *P* < 0.01; **Figure 5**). Microscopic analysis found that early stage SRC was associated with more depressed type than NSRC (OR = 1.31, 95%CI = 1.03–1.66, *P* < 0.05; **Figure 6A**). Moreover, an increased number of incidences with Borrmann type IV at the advanced stage was noted in SRC patients than that in NSRC patients (OR = 1.96, 95%CI = 1.45–2.66, *P* < 0.01; **Figure 6B**). However, no marked difference in LNM among advanced-stage SRC and NSRC was found (OR = 1.15, 95%CI = 0.74–1.80, *P* = 0.53, **Figure 7C**); while in all the GC cases or EGC cases, SRC was associated with less LNM, in comparison with NSRC (total: OR = 0.78, 95%CI = 0.63–0.96, *P* < 0.01; EGC: OR = 0.64, 95%CI = 0.52–0.79, *P* < 0.01; **Figures 7A, B** **)**. Moreover, 10 studies employed chemotherapy, while no marked difference in the chemotherapy rate was found between SRC and NSRC (OR = 0.95, 95%CI = 0.70–1.27, *P* = 0.85; **Figure 8**).

### Prognosis

**Figure 9:**
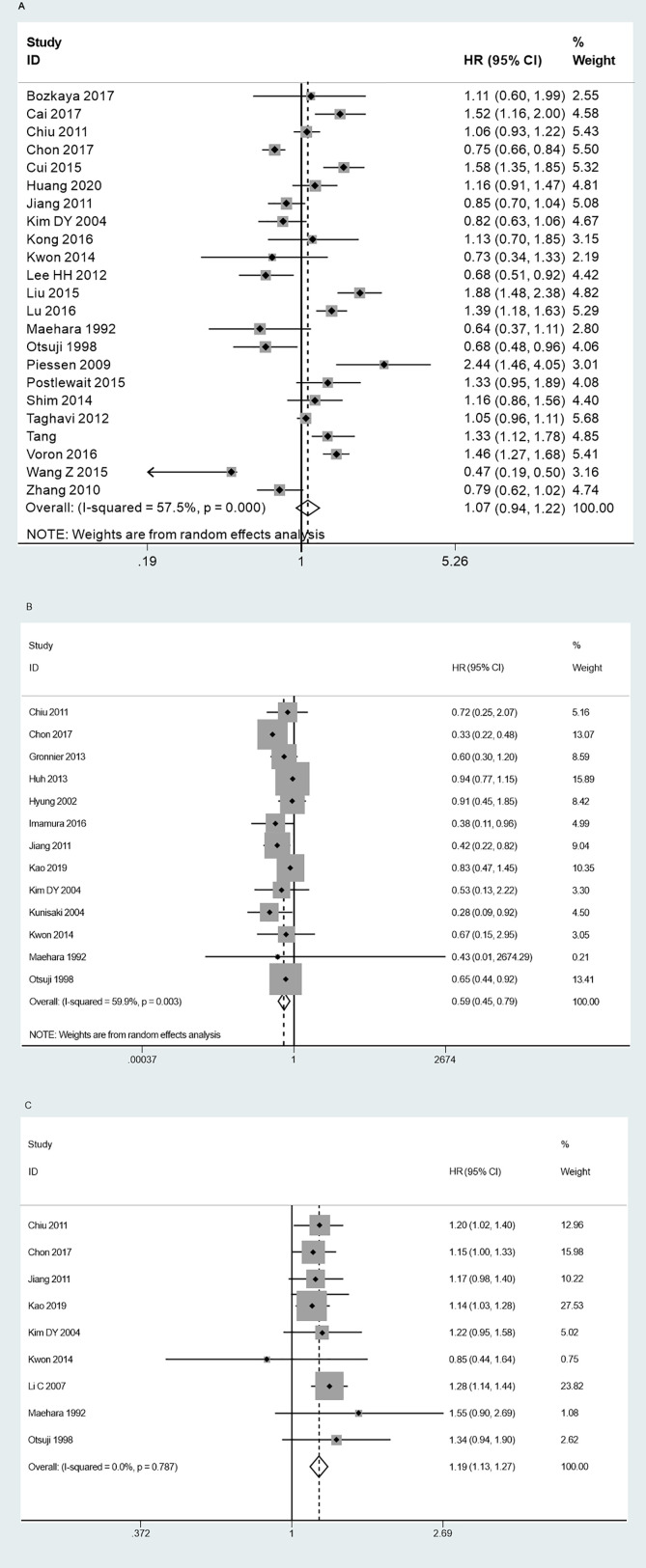
Forest plot displaying the results of meta-analysis. **(A)** Hazard ratio for overall survival of patients. **(B)** Hazard ratio for overall survival at early stage. **(C)** Hazard ratio for overall survival at advanced stage.

No statistically significant difference was noted in the overall survival between SRC and NSRC patients (HR = 1.07, 95%CI = 0.94–1.22, *P* = 0.285; **Figure 9A**). Early stage SRC exhibited better prognosis than NSRC (HR = 0.59, 95%CI = 0.45–0.79, *P* < 0.01; **Figure 9B**), while advanced-stage SRC exhibited poorer prognosis than NSRC (HR = 1.19, 95%CI = 1.13–1.27, *P* < 0.01; **Figure 9C**).

### Publication Bias

**Figure 10:**
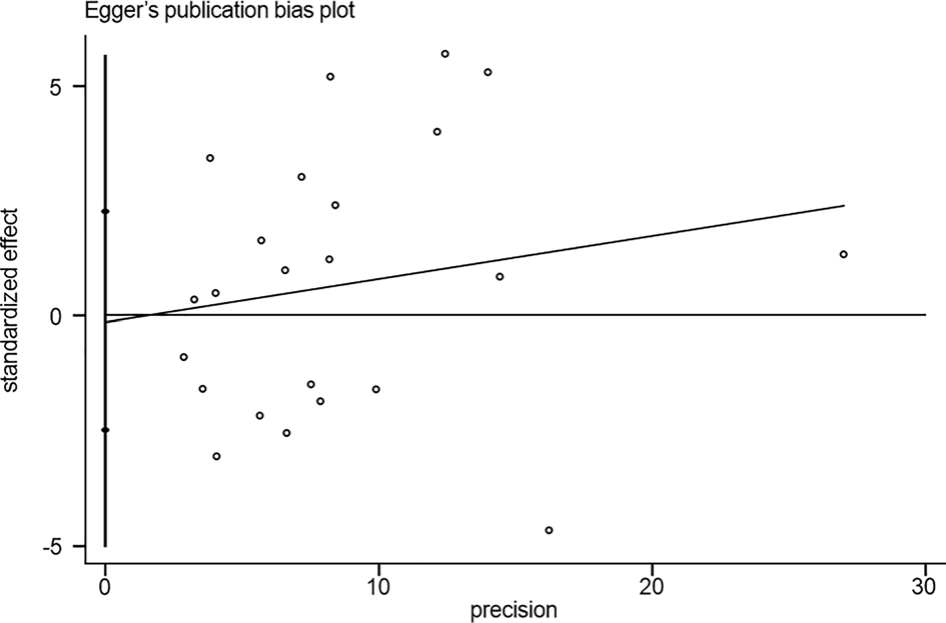
Test for publication bias. Egger’s test.

No noticeable publication bias was observed based on the results of Egger’s test (*P* = 0.416; **Figure 10**).

## Discussion

SRC is a highly malignant carcinoma mucocellulare. Abundant mucin in the cytoplasm of SRC drives the nuclei to one side of the cells, inducing a ring-like cell conformation (9). Approximately 1% of SRC occurs in organs including colon, urinary tract, gallbladder, pancreas, breast, and stomach. Previous studies have shown that SRC accounts for 8 to 30% of all gastric neoplasms (63). The global incidence of GC has recently declined, while that of gastric SRC is continually increasing (64). Although the included studies in this meta-analysis have reported the clinicopathological features of gastric SRC, the results are unclear.

Here, we found that gastric SRC was relatively frequently diagnosed in females; however, the underlying explanation for such an association has not yet been determined. Several studies have demonstrated the potential role of over-expressed estrogen in SRC, which has been associated with frequent metastasis in the uterus or ovary in SRC patients (65). Furthermore, Kim et al. found a substantially poorer overall survival in female SRC patients than in the male patients, especially those with advanced GC and aged ≤45 years (66). Here, the mean age of SRC patients was substantially younger than that of NSRC patients. The typical intracytoplasmic mucin, compressed nuclei in the corner, the tendency to be larger and sprawl superficially to mucosal and submucosal layers have ensured the early diagnosis of SRC at the early stage or younger age. Postlewait and Yokota claimed that the tumor size of NSRC was smaller than that of SRC. However, our study found no marked difference in the tumor size between the two groups (7, 9). Compared with NSRC, SRC was more commonly found in the middle location of the stomach; meanwhile, no marked difference between upper and lower locations was observed (data not shown). Thus, considering the macroscopic features of EGC, we suggested that SRC had more depressed type than NSRC. For AGC, Borrmann type IV was more commonly found in SRC than NSRC, which probably contributed to the poor outcome.

LNM is known to play a marked role in GC research. Our study implied that SRC was associated with less LNM than NSRC, especially for EGC, while no noticeable relationship was observed for AGC. Unlike other histological types, the correlation between the increased rate of LNM in SRC and the tumor size is not recognized (29). Due to the CDH1 mutation, early SRC was associated with a less aggressive state (67). SRC is thought to arise in the undifferentiated stem cell in lamina propria of gland neck. At the early stage, SRC was found to widely spread in the mucous layer and slowly to submucosal layer than NSRC. When SRC spread into the submucosa, it rapidly metastasized (68).Wang et al. reported that SRC was associated with less ulceration than NSRC, which was considered a major predictor for LNM (15).

The difference in prognosis between SRC and NSRC remains debatable. However, several studies have shown that SRC was associated with worse prognosis than NSRC (5, 23). However, Lee and Maehara reported the opposite results (10, 11). Our study indicated that the overall survival of SRC patients was insignificantly different from that of NSRC patients. The improved survival reported by several studies was probably related to the younger age of the SRC patients at presentation. Early stage SRC was associated with less LNM, and thus, it had better prognosis than NSRC. As a matter of fact, most of the included studies displayed that early stage SRC patients had a higher five-year survival rate. We extracted HR from the Kaplan–Meier curves, and the outcome was significant. Several studies have indicated that advanced-stage SRC was associated with poorer prognosis than NSRC (9, 50), while other studies could not find such an association (13, 34). The current study indicated that the poor prognosis of SRC was accompanied by the lower overall survival rate, as compared to NSRC. Consequently, early diagnosis and detection were crucial to improve the overall survival of gastric SRC. Furthermore, less invasive strategies, such as endoscopic submucosal dissection (ESD) and endoscopic mucosal resection (EMR), have been suggested for early stage GC to improve the quality of life of the patients. However, the Japanese GC treatment guidelines state that ESD was not feasible for the undifferentiated histology type of GC (69), which leads to controversial opinions on ESD for SRC treatment. Recent studies have reported dissimilar outcomes of ESD therapy for SRC (70). One study reported that ESD resulted in a higher rate of *en bloc* resection and complete resection on SRC than the poorly differentiated types of GC (35), which suggested that ESD might be preferred for EGC patients diagnosed with SRC. On the other hand, curative resection has been suggested for extended lymph node dissection for AGC. Furthermore, the effect of chemotherapy, either neoadjuvant of adjuvant, on SRC is still controversial (71, 72). Turgeon demonstrated that surgery resulted in a higher five-year overall survival rate than perioperative, neoadjuvant, and adjuvant therapy for stage I SRC patients (73). The chemosensitivity of SRC is related to the CLDN18-ARHGAP26/6 fusion (74). Compared with NSRC, SRC is speculated to be more chemo-resistant to conventional drugs, such as 5-FU and platinum (13, 23, 75). However, Pernot suggested that SRC possesses high sensitivity to taxane-based chemotherapeutic drugs or antiangiogenics (64). To improve the treatment strategy and prognosis is the important point of SRC.

This study has several limitations. First, most of the included studies were conducted in China, Korea, or Japan. The discrepancy in diet, heredity, and environment between Asia and other continents could have influenced the outcome. Second, no RCTs were included in this meta-analysis, and all the studies were retrospective analyses, which may result in a risk of bias. Third, since the HR was calculated from the data or extrapolated from the Kaplan–Meier curves, it was associated with reduced reliability. Finally, heterogeneity was high in the statistics process, which could lead to unavoidable biases. Therefore, additional data are essential to increase the quality and reliability of this meta-analysis.

## Conclusion

Gastric SRC is associated with more female patients, younger patients, more occurrence at middle location of the stomach, more depressed type (EGC), higher incidence of Borrmann type IV (AGC), and less LNM (EGC) than NSRC. The prognosis of early stage SRC is better than that of other GC types, while the prognosis of SRC at the advanced stage was poor. Thus, SRC exhibits specific biological features and differential prognosis compared with NSRC, which may facilitate the development of tailored therapeutic strategy and individualized treatment.

## Data Availability Statement

The original contributions presented in the study are included in the article/supplementary material. Further inquiries can be directed to the corresponding author.

## Author Contributions

SZ and C-GJ designed this study. SZ, LL, and KZ performed search and collected data. J-CZ and C-GJ re-checked the data. SZ and YT performed analysis. LL and SZ wrote the manuscript. C-GJ reviewed the manuscript. All authors contributed to the article and approved the submitted version.

## Funding

This work was supported by the General Project of Liaoning Provincial Education Department (JCZR2020004).

## Conflict of Interest

The authors declare that the research was conducted in the absence of any commercial or financial relationships that could be construed as a potential conflict of interest.

## Abbreviations

SRC, signet-ring cell carcinoma; NSRC, non signet-ring cell carcinoma; OR, odds ratio; HR, hazard ratio; CI, confidence interval; UICC, Union for International Cancer Control; GC, gastric cancer; EGC, early gastric cancer; AGC, advanced gastric cancer; LNM, lymph node metastasis.
